# Non-perforation tension pneumoperitoneum resulting from primary non-aerobic bacterial peritonitis in a previously healthy middle-aged man: a case report

**DOI:** 10.1186/s13256-016-0945-0

**Published:** 2016-06-06

**Authors:** Ognyan Georgiev Milev, Plamen Cekov Nikolov

**Affiliations:** Surgical Department, St Petka Multiprofile Hospital for Active Treatment, 119 Tsar Simeon Veliki St, 3700 Vidin, Bulgaria

**Keywords:** Pneumoperitoneum, Tension, Non-perforation, Peritonitis, Anaerobic, Primary

## Abstract

**Background:**

Tension pneumoperitoneum is a rare surgical emergency in which free intraperitoneal gas accumulates under pressure. The known sources of free gas are perforated hollow viscera. We believe this is the first published case of a tension non-perforation pneumoperitoneum secondary to anaerobic gas production. This occurred in a background of primary non-aerobic bacterial peritonitis, which developed in an immunocompetent adult man.

**Case presentation:**

A previously healthy 45-year-old Bulgarian man presented with a 3-week history of abdominal pain. He displayed signs of shock, peritonitis, and abdominal compartment syndrome. A plain abdominal X-ray showed the pathognomonic “saddlebag sign” with his liver displaced downwards and medially. An emergency laparotomy released pressurized gas, accompanied by 3100 mL of foamy pus. A sudden hemodynamic deterioration occurred soon after decompression. The sources of infection and tension pneumoperitoneum were not found. The peritoneal exudate sample did not recover aerobes. A laparostomy was created and three planned re-operations were performed. During the second re-laparotomy we placed an intraperitoneal silo and his abdomen was closed with skin sutures. Definitive fascial closure was achieved through separation of his two rectus muscles from their posterior sheaths. He was discharged in good health on the 25th postoperative day.

**Conclusions:**

Our case provides evidence supporting the theory that anaerobic infection may underlie the etiology of tension pneumoperitoneum. Prior to decompressive laparotomy the patient should receive an intravenous volume bolus to compensate for possible hypotension. If laparostomy leads to lateralization of the rectus muscles with a gap of 6 cm or less, the posterior part of the components separation technique is effective in achieving fascial closure. We present an original classification of tension pneumoperitoneum defining it as primary or secondary.

**Electronic supplementary material:**

The online version of this article (doi:10.1186/s13256-016-0945-0) contains supplementary material, which is available to authorized users.

## Background

Tension pneumoperitoneum (TP) is the accumulation of free gas under pressure within the peritoneal cavity leading to abdominal compartment syndrome (ACS). The latter is manifested by respiratory failure, which is due to compression of the diaphragm, and obstructive shock, which is the result of compression of the intra-abdominal veins. Compensatory peripheral vasoconstriction can maintain perfusion of vital organs for a limited time. It is pertinent that this situation may be a precursor to an unexpected cardiorespiratory arrest. The diagnosis is based on the clinical signs and symptoms, supported by a plain abdominal X-ray. Radiological findings include pneumoperitoneum, diaphragmatic elevation, downward and medial displacement of the liver (saddlebag sign), with centralization of bowel loops [1]. If TP occurs suddenly, emergency needle decompression is required. It should be followed by laparotomy if gastrointestinal rupture is suspected [2].

Seven electronic libraries were browsed to identify English language reports on TP. The period was from 1919 (introduction of abdominal X-ray) until 2015. The search revealed 124 publications with 159 cases of TP. A reference of significance was made by Singer to three German authors (1913 to 1914) who hypothesized that free gas accumulation could be due to gas-producing microorganisms [3]. This theory did not evolve; it was superseded by perforation of a hollow organ and one-way valve explanation of gas entry.

Our review revealed that all reported cases were secondary to perforations of hollow viscera. The digestive and respiratory systems were primary locations, with one case originating from the bladder. Of the 159 cases, 127 occurrences of TP were secondary to gastrointestinal perforations caused by: iatrogenic injury, 64; disease, 42; barotrauma and trauma, 11; and surgery, 10. Thirty one cases followed respiratory perforations caused by: iatrogenic injury, 4; barotrauma, 20; blunt chest trauma, 6; and surgery, 1. One case followed a bladder perforation caused by a Foley catheter left open to air. This analysis revealed that the incidence of TP resulting from gastrointestinal perforations was four times higher than those caused by ruptured respiratory organs (Additional file 1: Table S1).

We present a case of TP without perforation of a hollow organ. It is without similar reference within the English language literature over a 97-year period.

## Case presentation

A 45-year-old Bulgarian man presented with a vague abdominal pain. The onset of the pain was gradual and had persisted for 3 weeks. He had no comorbidities or surgeries, and did not take medication. His skin was pale, cold, and clammy. His breathing was labored with rapid and shallow respirations. Auscultation of his lungs revealed diminished breath sounds bibasally. His abdomen was bloated, diffusely tender, with guarding and rebound tenderness. Tympanism centrally and dullness laterally were noted on percussion. Bowel sounds were absent.

His vital signs on admission were: pulse, 125 minute; blood pressure, 80/40; respiratory rate, 38 minute; oxygen saturation on air, 90 %; and axillary temperature, 35.8 °C.

**Fig. 1:**
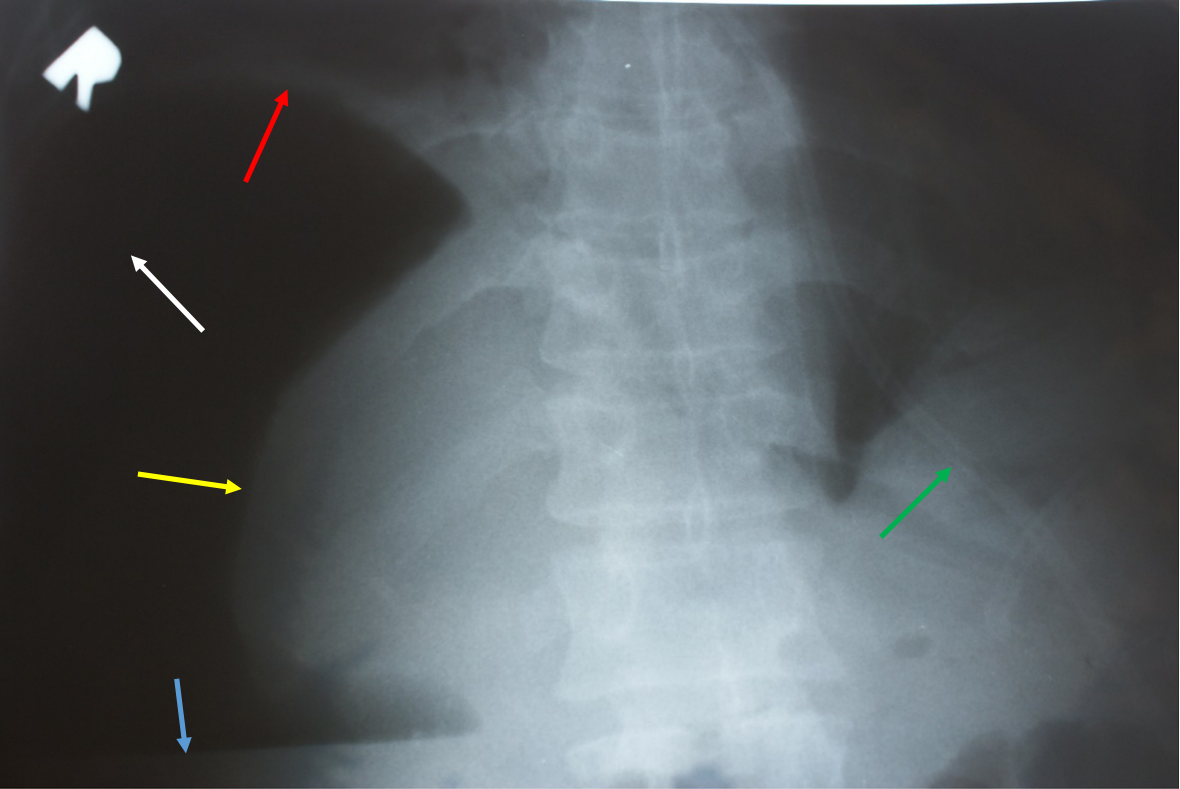
Erect plain abdominal X-ray on admission confirming tension pneumoperitoneum. Large amount of free gas under the right dome of the diaphragm, enveloping the liver (*white arrow*). The liver is diminished in size and has shifted downwards and medially, resembling a collapsed lung in pneumothorax: “the saddlebag sign” (*yellow arrow*). Liquid level in the free peritoneal cavity (*blue arrow*). High position of the right dome of the diaphragm (*red arrow*). Nasogastric tube (*green arrow*)

His intra-abdominal pressure (IAP) measured via urethral catheter was 26 mmHg. This was a grade IV intra-abdominal hypertension (IAH). The amount of urine on catheterization was 50 mL. Blood tests showed anemia, leucocytosis, azotemia, hypoproteinemia, and metabolic acidosis; alpha-amylase was normal. An erect plain X-ray of his abdomen revealed the saddlebag sign (Fig. 1). A chest X-ray disclosed the high position of his diaphragmatic domes, and a small reactive pleural effusion to the right.

He was in shock, with signs of generalized peritonitis and ACS. He received a short course of intravenous fluid resuscitation and broad-spectrum antibiotics. This was immediately followed by a midline laparotomy. Upon entering his peritoneal cavity, pressurized gas with a “rotten-egg” odor escaped and 3100 mL of frothy fetid pus evacuated. Despite preoperative rehydration, decompression of the abdomen resulted in sudden hypotension.

All of his organs and tissues were uniformly inflamed with no apparent focal site. His intestines were gathered medially in a ball and covered by a “shield” of dense fibrin deposits below which they appeared normal with shiny serosa and non-swollen walls. The appendicular end was indistinguishable from his small bowel loops, and beneath the fibrin shield looked normal. An appendectomy was performed.

Systematic exploration of all intraperitoneal, retroperitoneal organs and spaces (including the lesser sac) did not reveal the source of infection. Air-leak testing excluded a ruptured hollow viscus. A sealed perforation was also ruled out. Therefore we concluded that we were dealing with a primary bacterial peritonitis (PBP) of an anaerobic microorganism whose metabolism was responsible for the TP.

**Fig. 2:**
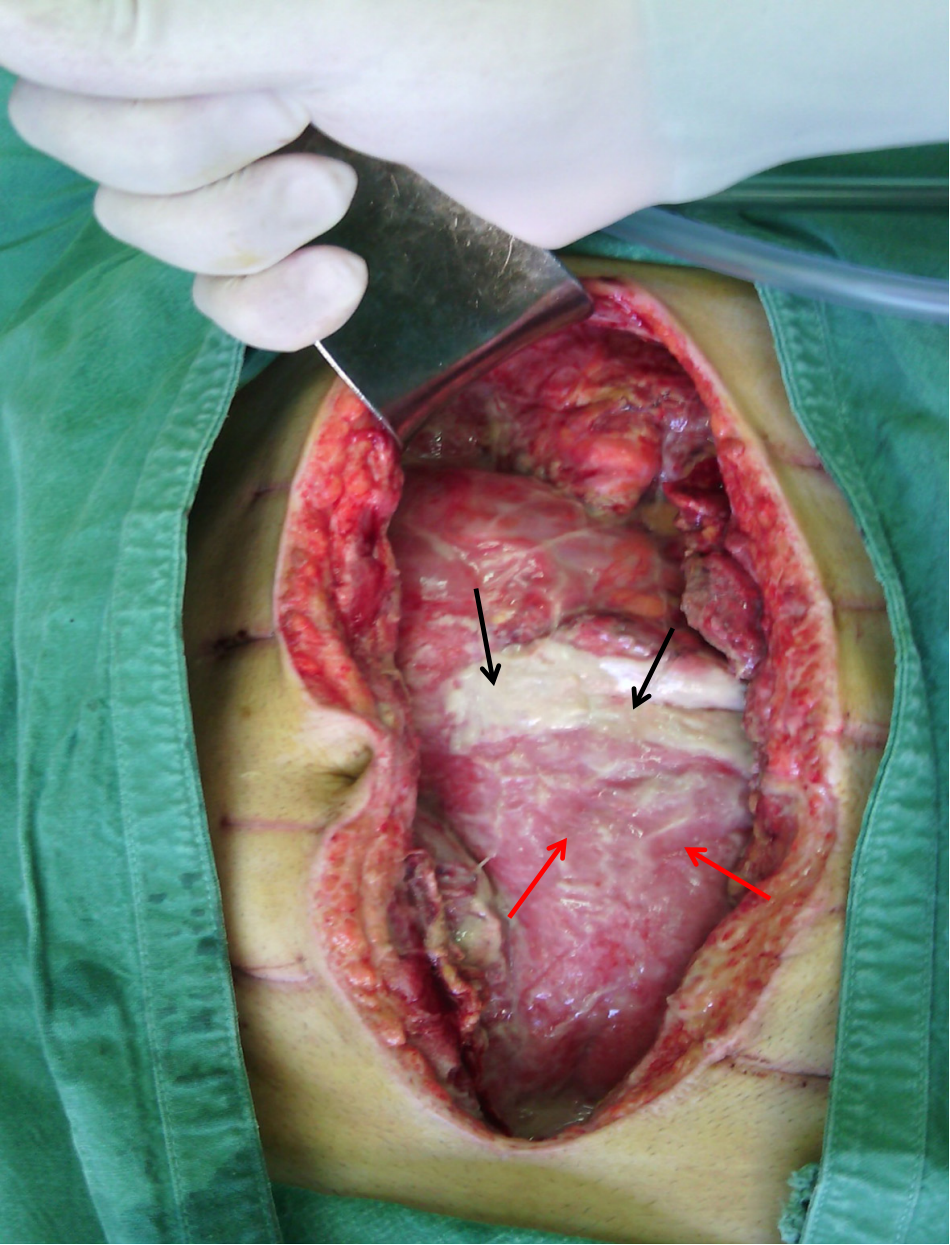
Second re-laparotomy 4 days after the index operation (picture taken from the patient’s legs). The fibrin “shield” which covered the abdominal organs on the index laparotomy has almost been absorbed, except for the area identified (*black arrows*). The latter gives a good idea of how the whole abdomen looked on the initial opening. The intestinal loops are already clearly visible (*red arrows*)

His abdomen was washed out and left open. His IAP was monitored during the first 48 hours, and fluctuated between 10 and 15 mmHg. Three planned re-laparotomies were performed at 48-hour intervals for debridement, wash out, and exploration for any septic source; none were found. At the end of each re-entry, the laparostomy was partially closed with fascial sutures at both ends. Rapid degradation of the fibrin shield made the bowel loops distinguishable on the second re-entry (Fig. 2). On this occasion there was no danger of ACS with IAP <10 mmHg and no organ dysfunction. Consequently we used the intraperitoneal silo technique by placing a presterilized sheet of cellophane over his intestines. It was tucked into the lateral paracolic gutters, and his abdomen was closed using only skin sutures.

On the third re-laparotomy, the silo was removed and the decision was made to definitively close his abdomen. Fascial suture was not possible because the central section remained with an insurmountable gap of 6 cm. We performed the posterior part of the components separation technique (CST) described by Ramirez *et al.* [4]. Longitudinal incisions of the two posterior rectal sheaths parallel and near his linea alba were made. We bluntly separated these sheaths from his rectus muscles, paying attention not to disturb the neurovascular bundles at the lateral rectal borders. This led to a good mobilization of the two rectal myofascial complexes (RMFCs), allowing apposition under acceptable tension. We used the Kloppel interrupted suture technique, and placed four Smead-Jones internal retention sutures. Our patient made an uneventful recovery, with the wound healing by primary intention.

The sample of the peritoneal exudate for culture and sensitivity taken during the initial operation did not grow bacteria. The samples from the three re-operations as well as the urine culture recovered *Acinetobacter baumannii (anitratus*). Blood samples were sterile. This bacterium is a Gram-negative obligate aerobe that usually causes nosocomial infections that are rarely clinically significant. With sensitivity only to carbapenems, a 6-day course of imipenem (1.5 g/day) led to complete eradication. The exudate samples for biochemical testing for bilirubin and alpha-amylase revealed levels corresponding to non-elevated serum samples.

A histological examination of the removed appendix showed that its end was reactively inflamed from the outside in. The mucosa was intact, as was the proximal appendicular wall through its full thickness. This excluded the appendix as the primary source of the infection.

Our patient was negative for HIV, hepatitis B, and hepatitis C. He had normal levels of immunoglobulins and was immunocompetent. Before discharge, the following imaging tests were performed: (a) a follow-through with liquid contrast, which revealed no structural or transit-times abnormalities throughout his gastrointestinal tract; (b) a computed tomography of his abdomen, which showed normal findings for all parenchymal and hollow organs.

He was discharged in good health 25 days after admission. He had no complaints at the 1- month and 6-month follow-up. He gained 10 kg in weight and returned to work. His abdominal wall was healthy; an abdominal ultrasound and chest X-ray were normal. A tuberculosis screening test was negative. He continued to be symptom-free with normal findings on physical examination 18 months after discharge.

## Discussion

The main characteristic aspect of the TP in our case was its slow development. This avoided the need for abdominal paracentesis and determined the clinical presentation. The slow progression of the disease allowed the accumulation and subsequent compression of free gas from anaerobic respiration. In addition, this enabled the mechanical processes of the TP to overlap the immune responses to peritonitis. This gradual increase in IAP provided time for the respiratory and cardiovascular compensatory reserves to intervene. Consequently, the clinical signs were less dramatic compared to cases of TP described in the literature search.

Normally, decompression of the abdomen results in rapid hemodynamic stabilization. Although rare, abdominal decompression can cause further hemodynamic deterioration. An immediate drop in IAH can “steal” blood flow, diverting it to the splanchnic area, and reducing heart preload to critical levels. This effect and concomitant decrease in systemic vascular resistance result in a sudden drop in aortic pressure. Reperfusion injury may occur and lead to extreme hyperkalemia and possible cardiac arrest. Therefore, shortly before decompression, the patient should receive an intravenous volume bolus [5]. If hypotension should occur, compression of the aorta at the diaphragmatic hiatus can reduce the vascular bed while the anesthesiologists restore intravascular volume [6].

We were unable to isolate the causative agent of the primary peritonitis because the sample was taken using the traditional method for aerobes. The latest guidelines suggest that anaerobic cultures are not necessary for patients with community-acquired intra-abdominal infection if empiric antimicrobial therapy active against common anaerobic pathogens is provided [7]. The evidence that the causative agent in our case was an anaerobic bacterium was derived by exclusion of other microorganisms:

- The lack of bacterial growth in an apparent infectious peritonitis without previous antibiotic therapy suggests that an obligate anaerobic bacterium is the sole causative agent. It could not be a facultative anaerobic bacterium or a mixed infection because they would culture on standard agar medium in the presence of oxygen [8].
- The odor of rotten eggs is caused by hydrogen sulfide (H_2_S). This gas is one of the end products of anaerobic respiration and not fermentation. Fungi only use fermentation consequently they could not be the causative agent in this case. This also excludes other aerobes such as fastidious bacteria, mycobacteria, and viruses, none of these produce H_2_S. Note that certain aerobic sulfate-reducing bacteria such as *Desulfovibrio* organisms can produce H_2_S, but they would recover on standard agar medium [9].
- The rapid improvement in the local findings noticed at the first and second re-laparotomies would only occur if immediate eradication was achieved. This could only take place if an obligate anaerobic bacterium was the sole cause of the infection and then “killed” by the oxygen; antibiotics could not do this within 48 hours [8].

The clinical features of the case required a laparostomy instead of primary abdominal closure [10]. When a laparostomy is no longer needed but the abdomen would benefit from further debridement, intraperitoneal silo is a viable interim procedure. It was described by Steinberg in 1979 and further developed by Fernandez *et al.* in 1999 [11]. This technique facilitates early temporary closing of the abdomen with skin sutures, keeping the front abdominal wall free of bowel adhesions for final fascial closure.

The latest clinical statements recommend that abdominal fascial closure be made within same-hospital-stay [12]. This was completed on the sixth day after the laparostomy. Despite this early period and the implementation of progressive closure, primary fascial suture was not possible. The clinical statements do not offer clear guidance on the use of CST within the acute settings of closing a laparostomy. Our approach was a “titrated” posterior version of the CST. With this maneuver each RMFC can be advanced medially by 2 to 4 cm [13]. We believe that this approach is safe and efficient in acute settings, provided the gap, which prevents fascial apposition, is ≤6 cm.

PBP is extremely rare, comprising less than 1 % of all cases. It is usually monobacterial and develops as a complication of another comorbidity and/or in immunocompromised patients [14]. We searched the same seven electronic libraries and only found three cases of PBP in healthy patients caused by anaerobes: two teenagers and a 21-year-old woman (Additional file 1: Table S2).

Although rare, TP is well documented but without classification. Based on the source of gas, we use and present our original classification (Table 1):

- *“Primary tension pneumoperitoneum”* for those instances in which the cause is localized below the diaphragm, within the peritoneal cavity.
- *“Secondary tension pneumoperitoneum”* for cases in which gas originates above the diaphragm, outside the peritoneal cavity (mediastinum, lungs, and pleural spaces).

**Table 1:** The proposed new classification of tension pneumoperitoneum

Primary tension pneumoperitoneum		Secondary tension pneumoperitoneum
Free gas originates below the diaphragm, within the peritoneal cavity.		Free gas originates above the diaphragm, outside the peritoneal cavity: mediastinum, lungs, and pleural spaces.
Perforation TP	Non-perforation TP	Esophageal and respiratory perforations
Iatrogenic (endoscopy and CPR)	Anaerobic gas production (anaerobic bacterial peritonitis)	Iatrogenic (EGD, bronchoscopy and orotracheal intubation)
Disease		Barotrauma (mechanical ventilation and blast injuries)
Blunt abdominal trauma and barotrauma (blast injuries)		Blunt chest trauma
Surgery		Surgery

*CPR* cardiopulmonary resuscitation, *EGD* esophagogastroduodenoscopy, *TP* tension pneumoperitoneum

The case here was placed in group (a).

## Conclusions

The combination of TP without perforated hollow viscus and primary anaerobic bacterial peritonitis is an extremely rare scenario in emergency surgery. Our case study provides evidence supporting the infectious etiology of TP. Shortly before laparotomy, the patient should be overhydrated to avoid decompression hypotension. The existing TP in a background of severe abdominal sepsis necessitates laparostomy. The posterior part of the Ramirez operation can be safely utilized, providing the insurmountable gap is ≤6 cm. We classify TP as primary or secondary.

## Abbreviations

ACS, abdominal compartment syndrome; CST, components separation technique; H_2_S, hydrogen sulfide; HIV, human immunodeficiency virus; IAH, intra-abdominal hypertension; IAP, intra-abdominal pressure; PBP, primary bacterial peritonitis; RMFC, rectal myofascial complex; TP, tension pneumoperitoneum.

## Additional file

Additional file 1: Table S1. Sources of tension pneumoperitoneum in cases reported in the English medical literature for the period 1919–2015. **Table S2.** Cases of primary anaerobic bacterial peritonitis in healthy patients reported in the English medical literature. (DOCX 60 kb)
