# Supplementary material for: Non-perforation tension pneumoperitoneum resulting from primary non-aerobic bacterial peritonitis in a previously healthy middle-aged man: a case report
Source: J Med Case Rep. 2016 Jun 6;10:163. doi: 10.1186/s13256-016-0945-0 (PMC4893842; doi:10.1186/s13256-016-0945-0)
Supplement: Additional file 1: Table S1. — Sources of tension pneumoperitoneum in cases reported in the English medical literature for the period 1919–2015. Table S2. Cases of primary anaerobic bacterial peritonitis in healthy patients reported in the English medical literature. (DOCX 60 kb) [file 13256_2016_945_MOESM1_ESM.docx]

**Additional file 1**

| **Authors, Year, ^[ref]^** | **Case**  **No** | **Source of gas** | **Cause** |
| --- | --- | --- | --- |
|  |  |  |  |
| Singer H, 1932 ^[1]^  **(Falkenburg C, 1913; Fründ H, 1914; Coenen H, 1913)^[^*^subref]^ | 1  ^not^  ^known^ | Gastrointestinal  *Peritonitis* | Sigmoid diverticulum perforation  *Infection by a gas-producing microorganism* |
| Maddock WG et al,1941 ^[2]^ | 4 | Gastrointestinal | Bowel perforations |
| Ronald Jones, 1945 ^[3]^ | 1 | Respiratory | Bronchial tear |
| Conole F et al, 1952 ^[4]^ | 1 | Pharynx | Resection of pharyngeal diverticulum |
| Knight AM, 1961 ^[5]^ | 1 | Gastrointestinal | Emphysematous gastritis perforation |
| Hector A, 1968 ^[6]^ | 15 | Gastrointestinal | Digestive tract perforation with peritonitis |
| Lagundoyle SB et al, 1970 ^[7]^ | 2 | Gastrointestinal | Septic colonic micro perforations |
| Hall R, 1971 ^[8]^ | 2 | Gastrointestinal | Colostomy leak |
| Thiele BL, 1973 ^[9]^ | 2 | Gastrointestinal | Pneumatic colonic injury |
| Bender J, 1974 ^[10]^ | 1 | Gastrointestinal | Colostomy closure |
| Echave V et al, 1975 ^[11]^ | 1 | Gastrointestinal | Duodenal ulcer perforation |
| Ogg TW et al, 1975 ^[12]^ | 1 | Gastrointestinal | Perforation from OGD |
| Addison NV et al, 1976 ^[13]^ | 4 | Gastrointestinal | 3 Obstructive cecal perforations, 1 Duodenal ulcer perforation |
| Irwin T, 1976 ^[14]^ | 1 | Gastrointestinal | Anastomotic leak |
| Linch D et al, 1979 ^[15]^ | 1 | Gastrointestinal | Gastric rupture from CRP |
| Gimmon Z et al, 1980 ^[16]^ | 1 | Gastrointestinal | Ischemic colitis perforation |
| Hutchinson GH et al,1980 ^[17]^ | 1 | Gastrointestinal | Gastric rupture from aerophagy |
| Rockahr GJ et al, 1980 ^[18]^ | 1 | Gastrointestinal | Gastric rupture from faulty nasal application of oxygen |
| Taub SJ et al, 1980 ^[19]^ | 1 | Gastrointestinal | Gastric ulcer perforation from OGD |
| Roberts RB et al, 1981 ^[20]^ | 1 | Gastrointestinal &  Respiratory | Gastric rupture with mechanical ventilation |
| Mills SA et al, 1983 ^[21]^ | 1 | Gastrointestinal | Gastric rupture after oesophageal intubation in CPR |
| Olinde AJ et al, 1983 ^[22]^ | 1 | Gastrointestinal | Gastric ulcer perforation |
| Ehrlich CP et al, 1984 ^[23]^ | 1 | Gastrointestinal | Colonic perforation from colonoscopy |
| Ballet TH et al, 1985 ^[24]^ | 1 | Gastrointestinal | Gastric rupture after oxygen flow into nasogastric tube |
| Biert J et al, 1987 ^[25]^ | 1 | Gastrointestinal | Gastric perforation after aerophagia |
| Diaz JH, 1987 ^[26]^ | 1 | Gastrointestinal | Repair of congenital diaphragmatic hernia |
| Higgins JRA et al, 1988 ^[27]^ | 1 | Gastrointestinal | Anastomotic leak |
| Ralston C et al, 1989 ^[28]^ | 1 | Respiratory | Barotrauma from mechanical ventilation |
| Yip A et al, 1989 ^[29]^ | 1 | Urinary | Bladder catheter left open to air |
| Cameron PA et al, 1991 ^[30]^ | 1 | Gastrointestinal | Gastric rupture from CPR |
| Yip AWC et al, 1991 ^[31]^ | 1 | Gastrointestinal | Colonic perforation from colonoscopy |
| Barnett T et al, 1992 ^[32]^ | 1 | Gastrointestinal | Colonic injury following colonoscopy |
| Winer-Muram HT et al,1993^[33]^ | 2 | Respiratory | Barotrauma from mechanical ventilation |
| Critchley LA et al, 1994 ^[34]^ | 1 | Respiratory | Barotrauma from mechanical ventilation |
| Schwarz RE et al, 1994 ^[35]^ | 2 | Respiratory | Barotrauma from mechanical ventilation |
| Serdyn C et al, 1994 ^[36]^ | 1 | Gastrointestinal | Gastric ulcer perforation |
| Lal AB et al, 1995 ^[37]^ | 1 | Respiratory | Barotrauma from mechanical ventilation and tracheal tear |
| Burdett-Smith et al, 1996 ^[38]^ | 1 | Respiratory | Barotrauma from positive pressure ventilation |
| Chan SY et al, 1996 ^[39]^ | 1 | Respiratory | Barotrauma from mechanical ventilation |
| Kealey WD et al, 1996 ^[40]^ | 1 | Gastrointestinal | Percutaneous endoscopic gastrojejunostomy |
| Suh HH et al, 1996 ^[41]^ | 2 | Gastrointestinal | Pneumatic colonic injury |
| Miller JS et al, 1997 ^[42]^ | 2 | Gastrointestinal | Gastric rupture from difficult endotracheal intubation |
| Oppenheim A et al, 1998 ^[43]^ | 2 | Respiratory | Pulmonary blast injury |
| Strear CM et al, 1998 ^[44]^ | 1 | Gastrointestinal | Gastric rupture from CPR |
| Ferrera P et al, 1999 ^[45]^ | 2 | Respiratory | Blunt chest trauma |
| Kim SJ et al, 2000 ^[46]^ | 1 | Gastrointestinal | Pneumatic colonic injury |
| Lau YS et al, 2000 ^[47]^ | 1 | Gastrointestinal | Gastric rupture from CPR |
| Llorens J et al, 2000 ^[48]^ | 1 | Respiratory | Barotrauma from mechanical ventilation |
| Devine JF et al, 2001 ^[49]^ | 1 | Gastrointestinal | Gastric rupture from vomiting |
| Khan Z et al, 2002 ^[50]^ | 2 | Gastrointestinal &  Respiratory | 1 Colo-coloanastomosis & 1 Pulmonary barotrauma from positive pressure ventilation |
| Ortega-C J et al, 2002 ^[51]^ | 1 | Gastrointestinal | Gastric rupture from OGD |
| Canivet JL et al, 2003 ^[52]^ | 1 | Respiratory | Barotrauma from mechanical ventilation |
| Luo CC et al, 2003 ^[53]^ | 1 | Oesophagus | Instrumental perforation of an obstructed esophagus in an infant |
| Pascu M et al, 2004 ^[54]^ | 1 | Gastrointestinal | Duodenal perforation from ERCP |
| Tsai L et al, 2004 ^[55]^ | 1 | Gastrointestinal | Colonic perforation after augmentation gastrocystoplasty |
| Ho CM et al, 2005 ^[56]^ | 1 | Gastrointestinal | Gastric rupture after awake fibreoptic intubation |
| Ayoob R et al, 2006 ^[57]^ | 1 | Respiratory | Barotrauma from mechanical ventilation |
| Dias LT et al, 2006 ^[58]^ | 1 | Gastrointestinal | Gastric rupture from CPR |
| Lee ES et al, 2006 ^[59]^ | 1 | Gastrointestinal | Colonic perforation from colonoscopy |
| Lu TC et al, 2006 ^[60]^ | 1 | Gastrointestinal | Gastric ulcer perforation from OGD |
| Richmond BK et al, 2006 ^[61]^ | 1 | Gastrointestinal | Duodenal perforation from OGD |
| Campillo-Soto A et al, 2007^[62]^ | 1 | Gastrointestinal | Gastric rupture from CPR |
| Filho WN et al, 2007 ^[63]^ | 1 | Respiratory | Barotrauma from mechanical ventilation |
| Hur H et al, 2007 ^[64]^ | 1 | Gastrointestinal | Colonic perforation from colonoscopy |
| Milanchi S et al, 2007 ^[65]^ | 1 | Gastrointestinal | Duodenal perforation from OGD |
| Sohoni A et al, 2007 ^[66]^ | 1 | Gastrointestinal | Pneumatic reduction of pediatric intussusception |
| Tam WY et al, 2007 ^[67]^ | 1 | Gastrointestinal | Duodenal ulcer perforation from OGD |
| Alder AC et al, 2008 ^[68]^ | 1 | Respiratory | Blunt chest trauma with tension pneumothorax and diaphragmatic tear |
| Boker AM, 2008 ^[69]^ | 1 | Respiratory | Tracheal tear from laser pediatric bronchoscopy |
| Gumpert R et al, 2008 ^[70]^ | 1 | Respiratory | Blunt chest trauma with tension pneumothorax and diaphragmatic tear |
| Hahn CD et al, 2008 ^[71]^ | 1 | Gastrointestinal | Gastric rupture from CPR |
| Keldahl M et al, 2008 ^[72]^ | 1 | Gastrointestinal | Gastric rupture from CPR |
| Sajith A et al, 2008 ^[73]^ | 1 | Gastrointestinal | Oesophagogastric rupture from CPR |
| Fernandes ML et al, 2009 ^[74]^ | 1 | Gastrointestinal | Colonic perforation from colonoscopy |
| Fu K et al, 2009 ^[75]^ | 1 | Gastrointestinal | Colonic injury from endoscopic submucosal dissection |
| Hassan N et al, 2009 ^[76]^ | 1 | Gastrointestinal | Duodenal perforation from ERCP |
| Jo GR et al, 2009 ^[77]^ | 1 | Gastrointestinal | Pneumatic colonic injury |
| Lin YT et al, 2009 ^[78]^ | 1 | Respiratory | Tracheal tear during orotracheal intubation |
| Mourissoux G et al, 2009 ^[79]^ | 1 | Gastrointestinal | Oesophagogastric rupture from transoesophageal ultrasonography |
| Gologorsky E et al, 2010 ^[80]^ | 1 | Respiratory | Intraoperative bronchial rupture |
| Lin BW et al, 2010 ^[81]^ | 1 | Gastrointestinal | Colonic perforation from colonoscopy |
| Morse JL et al, 2010 ^[82]^ | 1 | Gastrointestinal | Small bowel metastasis perforation |
| O'Hanlon KP, 2010 ^[83]^ | 1 | Gastrointestinal | Gastric rupture from CPR |
| Reichardt JA et al, 2010 ^[84]^ | 1 | Gastrointestinal | Gastric rupture from CPR |
| Siboni S et al, 2010 ^[85]^ | 1 | Gastrointestinal | Gastric injury from endoscopic submucosal dissection |
| Chiapponi C et al, 2011 ^[86]^ | 3 | Gastrointestinal | Colonic perforation from colonoscopy |
| Jones AE et al, 2011 ^[87]^ | 1 | Gastrointestinal | Perforated sigmoid diverticulum |
| Kim YI et al, 2011, ^[88]^ | 1 | Gastrointestinal  or Respiratory | Undiscovered source after CPR |
| Pilate S et al, 2011 ^[89]^ | 1 | Respiratory | Blunt chest trauma, tension pneumothorax and ipsilateral diaphragmatic rupture |
| Saber A, 2011 ^[90]^ | 1 | Gastrointestinal | Duodenal ulcer perforation |
| Akoglu H et al, 2012 ^[91]^ | 1 | Gastrointestinal | Gastric ulcer perforation |
| Bunni J et al, 2012 ^[92]^ | 1 | Gastrointestinal | Gastric barotrauma in a scuba diver |
| Carey JL et al, 2012 ^[93]^ | 1 | Gastrointestinal | Colonic perforation from colonoscopy |
| Hirose H et al, 2012 ^[94]^ | 1 | Respiratory | Barotrauma from extracorporeal membrane oxygenation |
| Hughes DB et al, 2012 ^[95]^ | 1 | Respiratory | Barotrauma from mechanical ventilation |
| Jalali SM et al, 2012 ^[96]^ | 1 | Gastrointestinal | Gastric rupture from CPR |
| Kalokhe A et al, 2012 ^[97]^ | 1 | Gastrointestinal | Colonic perforation from colonoscopy |
| Patel MB et al, 2012 ^[98]^ | 1 | Gastrointestinal | Traumatic gastric rupture |
| Prochazka R et al, 2012 ^[99]^ | 1 | Gastrointestinal | Air leakage during endoscopic gastric drainage of a pancreatic pseudocyst |
| Simeonidis N et al, 2012 ^[100]^ | 1 | Gastrointestinal | OGD induced duodenal ulcer perforation |
| Souadka A et al, 2012 ^[101]^ | 1 | Gastrointestinal | Colonic perforation from colonoscopy |
| Zotos PG et al, 2012 ^[102]^ | 1 | Respiratory | Barotrauma from mechanical ventilation |
| Dastidar А, 2013 ^[103]^ | 1 | Respiratory | Bronchus rupture from rigid bronchoscopy |
| Ergin M et al, 2013 ^[104]^ | 1 | Gastrointestinal | Pneumatic colonic injury |
| Fallon SC et al, 2013 ^[105]^ | 4 | Gastrointestinal | Colonic perforation from pneumatic reduction of pediatric intususception |
| Lawther S et al, 2013 ^[106]^ | 1 | Gastrointestinal | Neonatal gastric perforation from nasogastric feeding tube |
| Pourmand A et al, 2013 ^[107]^ | 1 | Gastrointestinal | Air leakage from colonoscopy without apparent rupture |
| Safi F et al, 2013 ^[108]^ | 1 | Respiratory | Ruptured pneumatocele from mechanical ventilation |
| Chen PN et al, 2014 ^[109]^ | 1 | Gastrointestinal | Gastric rupture after accidental oesophageal intubation |
| Consiglieri C et al, 2014 ^[110]^ | 1 | Gastrointestinal | Air leakage during endoscopic gastric drainage of a pancreatic pseudocyst |
| Di Saverio S et al, 2014 ^[111]^ | 1 | Gastrointestinal | Colonic perforation from colonoscopy |
| Montazeri М et al, 2014 ^[112]^ | 1 | Gastrointestinal | Pneumatic colonic injury |
| Monterrubio-V J et al,2014 ^[113]^ | 1 | Respiratory | Barotrauma from apnoea testing for brain death |
| Shashidhara P et al, 2014 ^[114]^ | 1 | Gastrointestinal | Pneumatic colonic injury |
| Ternlund SP et al, 2014 ^[115]^ | 1 | Gastrointestinal | Gastric ulcer perforation |
| Thatte М et al, 2014 ^[116]^ | 1 | Gastrointestinal | Pneumatic colonic injury |
| Webman R et al, 2014 ^[117]^ | 1 | Respiratory | Blunt thoracic trauma |
| Williams DT et al, 2014 ^[118]^ | 1 | Gastrointestinal | Gastric rupture from CPR |
| Chen YG et al, 2015 ^[119]^ | 1 | Gastrointestinal | Lung cancer bowel metastasis perforation |
| Kaafarani H. 2015 ^[120]^ | 1 | Gastrointestinal | Colonic perforation from colonoscopy |
| Miller D, 2015 ^[121]^ | 1 | Gastrointestinal | Cecal perforation from distal obstruction |
| Sy E et al, 2015 ^[122]^ | 1 | Gastrointestinal | Pneumatic colonic injury |
| Thornton S, 2015 ^[123]^ | 1 | Gastrointestinal | Gastric rupture from non-accidental trauma |
| Vishnu RM et al, 2015 ^[124]^ | 1 | Gastrointestinal | Gastric rupture from CPR |
| ***OGD:** oesophagogastroduodenoscopy; **CPR:** cardiopulmonary resuscitation; **ERCP:** endoscopic retrograde cholangio-pancreatography | | | |

1. **Table S1** Sources of tension pneumoperitoneum in cases reported in the English medical literature for the period 1919-2015.

**Searched electronic libraries:** Pubmedcentral, Willey online library, Cochrane Library, Elsevier, The Freeibrary, Google Scholar, Internet Scientific Publications.

See below for the reference list of this research.

1. Singer H. Valvular pneumoperitoneum. JAMA. 1932;99(26):2177-80.

*(*Falkenburg, C:* Ein Fall von Gasasammlung in der freien Bauchhöhle, Deutsche Ztschr. F. Chir. 124:130-136, 1913.  *Fründ, Heinrich:* Gasbildung in der freien Bauchhöhle, Deutsche Ztschr. F. Chir. 130:383-392, 1914. *Coenen, H.:* Der Gashrand. Die Gasperitonitis, chapter 24, Ergebn. D. Chir. u. Orthop. 11:130-136, 1913.) [subref]

1. Maddock WG, Coventry MB. A perfrated ulcer of the ileum opposite a Meckel’s diverticulum. Surg Gynec&Obst. 1941;73: 105.
2. Ronald Jones. Massive Surgical Emphysema, Pneumothorax, Pneumoperitoneum. Br Med J. 1945;2(4424):530–1.
3. Conole F, D’Angelo A. Resection of pharyngeal diverticulum with spontaneous development of tension pneumoperitoneum. Am J Surg. 1952;83(4):580-3.
4. Knight AM. Emphysematous gastritis and spontaneous tension pneumoperitoneum. J Med Assoc Ga. 1961;50:55-8.
5. Hector A. Pneumoperitoneum under tension in peritonitis due to digestive tract perforation (apropos of 15 cases). Actual Hepatogastroenterol. 1968;4(4):153-63.
6. Lagundoyle SB, Itayem SO. Tension pneumoperitoneum. Br J Surg. 1970;57:576-80.
7. Hall R. Delayed tension pneumoperitoneum after colostomy closure. Br J Surg. 1971;58:574-6.
8. Thiele BL. Compressed air injury of the colon: A report of two cases. Aust N Z J Surg. 1973;43:49-51.
9. Bender J. Tension pneumoperitoneum after resection of an anus praeternaturalis. Tijdschr Gastroenterol. 1974;17(4):253-8.
10. Echave V, Flores M, Ogilvy WL. Tension pneumoperitoneum: Case report and review of the literature. Can J Surg. 1975; 18:585-8.
11. Ogg TW, Davidson Al. Respiratory failure following gastroscopy. Anaesthesia. 1975;30:194-8.
12. Addison NV, Broughton AC. Tension pneumoperitoneum: A report of 4 cases. Br J Surg. 1976;63:877-80.
13. Irwin T. Postoperative tension pneumoperitoneum, a complication of colonic anastomosis: Report of a case. Dis Colon Rectum. 1976;19:68-70.
14. Linch D, McDonald A, McNicol L. Tension pneumoperitoneum complicating cardiac resuscitation. Intensive Care Med. 1979;5:93-4.
15. Gimmon Z, Berlatzky Y, Freund U. Tension pneumoperitoneum complicating nongangrenous nonocclusive ischemic colitis. Am J Proctol Gastroenterol Colon Rectal Surg. 1980;31:8.
16. Hutchinson GH, Alderson DM, Tumberg LA. Fatal tension pneumoperitoneum due to aerophagy. Postgrad Med J. 1980; 56:516-8.
17. Rockahr GJ. Stomach rupture and tension pneumoperitoneum after faulty nasal application of oxygen. Z Arztl Fortbild (Jena). 1980;74(8):376-8.
18. Taub SJ, Nagurka C, Raskin JB. Tension pneumoperitoneum as a nontraumatic complication of upper gastrointestinal endoscopy. Gastrointest Endosc. 1980;26:153-4.
19. Roberts RB, Blake BC, Bruggeman GE. Tension pneumoperitoneum-A cause of ventilatory obstruction. Anesthesiology. 1981;55:326-7.
20. Mills SA, Paulson D, Scott SM, Sethi G. Tension pneumoperitoneum and gastric rupture following cardiopulmonary resuscitation. Ann Emerg Med. 1983;12:94-5.
21. Olinde AJ, Carpenter D, Maher JM. Tension pneumoperitoneum: A cause of acute aortic occlusion. Arch Surg. 1983; 118:1347-50.
22. Ehrlich CP, Hall FM, Joffe N: Postendoscopic perforation of normal colon in an area remote from instrumentation-with secondary tension pneumoperitoneum. Gastrointest Endosc 1984;30:190-1.
23. Ballet TH, Michel L. Gastric rupture. A danger of postoperative oxygenation with a nasal catheter. Int Surg. 1985;70:265-6.
24. Biert J, ter Haar AM, Eggink WF. Tension pneumoperitoneum, an unusual complication of perforation of the stomach (Letter). Neth J Surg. 1987;39:134-5.
25. Diaz JH. Tension pneumoperitoneum-pneumothorax during repair of congenital diaphragmatic hernia. Anesth Analg. 1987; 66(6):577-80.
26. Higgins JRA, Halpin DMG, Midgley AK. Tension pneumoperitoneum: A surgical emergency. Br J Hosp Med. 1988; 39:160-1.
27. Ralston C, Clutton-Brock TH, Hutton P. Tension pneumoperitoneum. Intensive Care Med. 1989;15:532-3.
28. Yip A, Lau WY, Wong KK. Tension pneumoperitoneum: An unusual urologic cause. Br J Urol. 1989;64(2):199-200.
29. Cameron PA, Rosengarten PL, Johnson WR, Dziukas L. Tension pneumoperitoneum after cardiopulmonary resuscitation. Med J Aust. 1991;155:44-7.
30. Yip AWC, Chow WC, Chan J. Tension pneumoperitoneum after colonoscopic polypectomy (Letter). Endoscopy. 1991; 23:241.
31. Barnett T, McGeehin W, Chen C, Brennan E. Acute tension pneumoperitoneum following colonoscopy. Gastrointest Endosc. 1992;38:99-100.
32. Winer-Muram HT, Rumbak MJ, Bain RS. Tension pneumoperitoneum as a complication of barotrauma. Crit Care Med. 1993;21:941-3.
33. Critchley LA, Rowbottom S. Fatal tension pneumoperitoneum with pneumothorax. Anaesth Intensive Care. 1994;22:298-9.
34. Schwarz RE, Pham SM, Bierman MI, Lee KW, Griffith BP. Tension pneumoperitoneum after heart-lung transplantation. Ann Thorac Surg. 1994;57:478-81.
35. Serdyn C, Lake APJ. Tension pneumoperitoneum. Anesth Intensive Care. 1994;22:626-7.
36. Lal AB, Kumar N, Sami KA. Tension pneumoperitoneum from tracheal tear during pharyngolaryngoesophagectomy. Anesth Analg. 1995;80:408-9.
37. Burdett-Smith P, Jaffey L.Tension pneumoperitoneum. Accid Emerg Med. 1996;13:220-1.
38. Chan SY, Kirsch CM, Jensen WA, Sherck J. Tension pneumoperitoneum. West J Med.1996;165:61-4.
39. Kealey WD, McCallion WA, Boston VE. Tension pneumoperitoneum: a potentially life-threatening complication of percutaneous endoscopic gastrojejunostomy. J Pediatr Gastroenterol Nutr. 1996;22(3):334-5.
40. Suh HH, Kim YJ, Kim SK. Colorectal injury by compressed air--a report of 2 cases. J Korean Med Sci. 1996;11(2):179-82.
41. Miller JS, Itani KM, Oza MD, Wall MJ. Gastric rupture with tension pneumoperitoneum: a complication of difficult endotracheal intubation. Ann Emerg Med. 1997;30(3):343-6.
42. Oppenheim A, Pizov R, Pikarsky A, Weiss YG, Zamir G, Sprung CL. Tension pneumoperitoneum after blast injury: dramatic improvement in ventilatory and hemodynamic parameters after surgical decompression. J Trauma. 1998; 44(5):915-7.
43. Strear CM, Jarnagin WR, Schecter W, Mackersie RC, Hickey MS. Gastric rupture and tension pneumoperitoneum complicating cardiopulmonary resuscitation: case report. J Trauma. 1998;44(5):930-2.
44. Ferrera P, Chan L. Tension pneumoperitoneum caused by blunt trauma. Am J Emerg Med. 1999;17(4):351-3.
45. Kim SJ, Ahn SI, Hong KC, Kim JS, Shin SH, Woo ZH. Pneumatic colonic rupture accompanied by tension pneumoperitoneum. Yonsei Med J. 2000;41(4):533-5.
46. Lau YS, Kam CW. Tension pneumoperitoneumafter cardiopulmonary resuscitation. 2000; Hong Kong j.emerg.med. 2000; 7:110-3.
47. Llorens J, Martinez C, Sanz JC, Tarazona E, Sáez P, Tejados J. Tension pneumoperitoneum as complication in a case of perioperative barotrauma. Rev Esp Anestesiol Reanim. 2000;47(3):130-3.
48. Devine JF, McCarter TG Jr. Images in clinical medicine: tension pneumoperitoneum. N Engl J Med. 2001;344(26):1985.
49. Khan Z, Novell J. Conservative management of tension pneumoperitoneum. Ann R Coll Surg Engl. 2002;84:164-5.
50. Ortega-Carnicer J, Ruiz-Lorenzo F, Ceres F. Tension pneumoperitoneum due to gastric perforation. Resuscitation. 2002; 54(2):215-6.
51. Canivet JL, Yans T, Piret S, Frere P, Beguin Y. Barotrauma-induced tension pneumoperitoneum. Acta Anaesthesiol Belg. 2003;54(3):233-6.
52. Luo CC, Kong MS, Chao HC, Wu WJ. Tension pneumoperitoneum following instrumental perforation of an obstructed esophagus in an infant. Chang Gung Med J. 2003;26(10):768-71.
53. Pascu M, Hanke B, Wiedenmann B, Martens F, Dignass AU. Complication of Waldenström’s macroglobulinaemia following ERCP. Gut. 2004;53(12):1793.
54. Tsai L, Hi T, Long P. An Unusual Case Of Tension Pneumo-Peritoneum Causing Tension Pneumothorax And

Pneumomediastinum After Augmentation Gastrocystoplasty. The Internet Journal of Surgery. 2004;6(2).

1. Ho CM, Yin IW, Tsou KF, Chow LH. Gastric rupture after awake fibreoptic intubation in a patient with laryngeal carcinoma. Br. J. Anaesth. 2005:94(6):856-8.
2. Ayoob R, Subit M, Richmond B. Massive Pneumoperitoneum From Pulmonary Barotrauma: a Rare Complication, HCP Live. 2007. <http://www.hcplive.com/journals/resident-and-staff/2006/2006-06/2006-06_02> Accessed 10 Jan 2016.
3. Dias LT, Mendes LC, Mello PM, Santos LG, Vasconcelos JT. Gastric rupture following cardiopulmonary resuscitation: case report. Rev Bras Ter Intensiva. 2006;18(2):207-11.
4. Lee ES, Jang MK, Park SY, Lee JH, Lee JY, Lim EJ et al. A case of iatrogenic tension pneumoperitoneum following colonoscopy in a patient with cytomegalovirus colitis. Korean J Gastroenterol. 2006;47(4):312-5.
5. Lu TC, Chen SY, Wang HP, Lee CC, Chen SC. Tension pneumoperitoneum following upper gastrointestinal endoscopy. J Formos Med Assoc. 2006;105(5):431-3.
6. Richmond BK, Mullins B, Jackson M, Dyer B. Agarwal S. Tension pneumoperitoneum resulting from endoscopic duodenal perforation: a case report. W V Med J. 2006;102(6):26-7.
7. Campillo-Soto A, Lirón-Ruiz R, Torralba-MartinezJA, Morales-Cuenca G, del Pozo P, Aquavo-Albasini JL. Gastric rupture and massive pneumoperitoneum after cardiopulmonary resuscitation by lay persons. Cir Esp. 2007;81(1):49-51.
8. Filho WN, Cardenas S, da Paz V. Lone tension pneumoperitoneum associated to mechanical ventilation with hemodynamic instability. Crit Care. 2007;11 Suppl 3:104.
9. Hur H, Yang KM, Chung NE, Kang SM. A Case of Tension Pneumoperitoneum as a Fatal Complication of Colonoscopy. Korean J Leg Med. 2007;31(1):113-6.
10. Milanchi S, Margulies DR, Nissen NN. Tension Pneumoperitoneum: Management of a Surgical Emergency. HCP Live. 2007. http://www.hcplive.com/publications/surgical-rounds/2007/2007-03/2007-03_07 Accessed 08 Jan 2016.
11. Sohoni A, Wang NE, Dannenberg B. Tension pneumoperitoneum after intussusception pneumoreduction. Pediatr Emerg Care. 2007;23(8):563-4.
12. Tam WY, Bertholini D. Tension pneumoperitoneum, pneumomediastinum, subcutaneous emphysema and cardiorespiratory collapse following gastroscopy. Anaesth Intensive Care. 2007;35(2):307-9.
13. Alder AC, Hunt JL, Thal ER. Abdominal compartment syndrome associated with tension pneumoperitoneum in an elderly trauma patient. J Trauma. 2008;64(1):211-2.
14. Boker AM. Bilateral tension pneumothorax and pneumoperitonium during laser pediatric bronchoscopy--case report and literature review. Middle East J Anaesthesiol. 2008;19(5):1069-78.
15. Gumpert R, Archan S, B Kügler B, Seibert FJ, Prause G. Pneumoperitoneum following tension pneumothorax in combination with traumatic rupture of the diaphragm mimicks intraabdominal bleeding.  Injury Extra, 2008;39(11):368-70.
16. Hahn CD, Choi YU, Lee D, Frizzi JD. Pneumoperitoneum due to gastric perforation after cardiopulmonary resuscitation: case report. A J Crit Care. 2008;17(4):386-8.
17. Keldahl M, Sen S, Gamelli RL. Gastric rupture after cardiopulmonary resuscitation in a burn patient. Emerg Med J. 2008; 25(2):115-6.
18. Sajith A, O’Donohue, Roth RM, Khan RA. CT scan findings in oesophagogastric perforation after out of hospital cardiopulmonary resuscitation. Emerg Med J. 2008;25(2):115-6.
19. Fernandes ML, Pires KCC, Chimelli PHB, Issa MRN. Abdominal compartment syndrome during endoscopic clamping of an intestinal perforation secondary to colonoscopy. Rev. Bras. Anestesiol. 2009;59(5): 614-7.
20. Fu K, Ishiakawa T, Yamamoto T, Kaji Y. Paracentesis for successful treatment of tension pneumoperitoneum related to endoscopic submucosal dissection. Endoscopy. 2009;41 Suppl 2:245.
21. Hassan N, Mohamed A, Masoodi I, Emran F, Wani H, Abukhater M. Tension Pneumothorax, Unusual Presentation of Post-ERCP Duodenal Perforation. Case Report and Literature Review. Internet J Surg. 2009;24(1).
22. Jo GR, Lee SY, Kim KH, Ahn YW, Huh GY. Colorectal Trauma by Compressed Air. Korean J Leg Med. 2009;33(1):57-9.
23. Lin YT, Zuo Z, Lo PH, Hseu SS, ChaNG wk, Chan KH, Yuan HB. Bilateral tension pneumothorax and tension pneumoperitoneum secondary to tracheal tear in a patient with relapsing polychondritis. J Chin Med Assoc. 2009;72(9):488-91.
24. Mourissoux G, Schlumberger S, De Lentdecker P, Fischler M. Fatal pneumoperitoneum caused by nasopharyngeal oxygen delivery after transoesophageal echocardiography for cardiac surgery. Acta Anaesthesiol Scand. 2009;53(9):1223-5.
25. Gologorsky E, Gologorsky A, Stahl K, Nguven DM, Pham SM. Tension pneumoperitoneum as the sole presentation of an intraoperative bronchial rupture. J Heart Lung Transplant. 2010;29(9):1078-9.
26. Lin B W, Thanassi W. Tension Pneumoperitoneum. Journal of Emergency Medicine . 2010;38(1):57-9.
27. Morse JL, Safdar B. Acute tension pneumothorax and tension pneumoperitoneum in a patient with anorexia nervosa. J Emerg Med. 2010;38(3):13-6.
28. O’Hanlon KP. Gastric rupture with pneumoperitoneum after mouth-to-nose breathing in an infant. J Emerg Med. 2010; 39(3):312-5.
29. Reichardt JA, Casey GD, Krywko D. Gastric Rupture from Cardiopulmonary Resuscitation or Seizure Activity? A Case Report. J Emerg Med. 2010;39(3):309-11.
30. Siboni S, Bona D, Abate E, Bonavina L.Tension pneumoperitoneum following endoscopic submucosal dissection of leiomyoma of the cardia. Endoscopy. 2010;42 Suppl 2:152.
31. Chiapponi C, Stocker U, Körner M, Ladurner R_._ Emergency percutaneous needle decompression for tension pneumoperitoneum. BMC Gastroenterol. 2011;11:48.
32. Jones AE, Godfrey D, Nash GF. Tension pneumoperitoneum: innovative decompression of this general surgical emergency. Surg Tech Develop. 2011;1(2):52-3.
33. Kim YI, Han SK, Park SW. Unexplained massive pneumoperitoneum following cardiopulmonary resuscitation. Hong Kong j.emerg.med. 2011;18(1):31-3.
34. Pilate S, De Clercq S. Tension pneumothorax and life saving diaphragmatic rupture: a case report and review of the literature. World J Emerg Surg. 2011;6:23.
35. Saber A. Uncommon presentation of perforated duodenal ulcer: A report of three different cases. Surg Chronicles. 2011; 16(1):42-5.
36. Akoglu H, Coban E, Guneysel O. Tension pneumoperitoneum complicated with tension pneumothorax in a patient with diaphragmatic eventration. BMJ Case Rep. 2012;doi:10.1136/bcr.01.2012.5512.
37. Bunni J, Bryson PJ, Higgs SM. Abdominal compartment syndrome caused by tension pneumoperitoneum in a scuba diver. Ann R Coll Surg Engl. 2012;94(8):237-9.
38. Carey JL, Napoli AM. Tension pneumoperitoneum during routine colonoscopy. Am J Emerg Med. 2012;30(1):261.
39. Hirose H, Cavarocchi NC, Holoyda K. Tension pneumothorax on extracorporeal membrane oxygenation leading to significant pneumoperitoneum. Open Cardiovascular and Thoracic Surgery Journal. 2012;5:31-4.
40. Hughes DB, Judge TN, Spigland NA. Tension pneumoperitoneum in a child resulting from high-frequency oscillatory ventilation: a case report and review of the literature. J Pediatr Surg. 2012;47(2):397-9.
41. Jalali SM, Emani-Razavi H, Mansouri A. Gastric perforation after cardiopulmonary resuscitation. Am J Emerg Med. 2012; doi: 10.1016/j.ajem.2011.12.032.
42. Kalokhe A, Jacob J. Acute dyspnea after colonoscopy. Am J Med Sci. 2012; 343(4):327.
43. Patel MB, High K, Eckert M. Tension pneumoperitoneum after traumatic gastric rupture. Am Surg. 2012;78(9):435-6.
44. Prochazka R, Vidales G, Villa-Gómez G, Illescas A, Pereira N. Tension pneumoperitoneum as a complication of endoscopic ultrasound guided transgastric drainage of pancreatic pseudocyst: case report and review of the literature. Rev Gastroenterol Peru. 2012;32(1):88-93.
45. Simeonidis N, Ballas K, Pavlidis E, Psarras K, Pavlidis T, Sakantamis A. Tension Pneumoperitoneum: A Rare Complication of Upper Gastrointestinal Endoscopy. JSLS. 2012;16(3):495-7**.**
46. Souadka A, Mohsine R, Ifrine L, Belkouchi A, El Malki HO . Acute abdominal compartment syndrome complicating a colonoscopic perforation: a case report. J Medl Case Reports. 2012;6:51.
47. Zotos PG, Kontogiannis AG, Dimakopoulos AD, Tzamali EK.Tension pneumoperitoneum in association with tension pneumothorax. Am J Crit Care Med. 2012;186(12):1306.
48. Dastidar А. Pneumomediastinum, pneumoperitoneum and pneumothorax following iatrogenic perforation of bronchus: Successful conservative management of a potentially serious complication. J Pediatr Surg. 2013;48(2):456-8.
49. Ergin M, Ozer MR, Kocak S, Karakus N, Babagil B, Cander B. A Rare Case of Colorectal Injury With Compressed Air. J Med Cases. 2013;4(3):159-160.
50. Fallon SC, Kim ES, Naik-Mathuria BJ, Nuchtern JG, Cassady CI, Rodrigues JR. Needle decompression to avoid tension pneumoperitoneum and hemodynamic compromise after pneumatic reduction of pediatric intussusception. Pediatr Radiol. 2013;43(6):662-7.
51. Lawther S, Patel R, Lall A. Neonatal gastric perforation with tension pneumo-peritoneum. J Ped Surg Case Rep. 2013; 1(2):14-6.
52. Pourmand A, Shokoohi H. Tension Pneumothorax, Pneumoperitoneum, and Cervical Emphysema following a Diagnostic Colonoscopy. Case Rep Emerg Med. 2013; doi:10.1155/2013/583287.
53. Safi F, Siddiqui N, Valavoor S, Al-Natour M, Assaly R. Tension Pneumoperitoneum Complicating Tension Pneumothorax. Chest. 2013; doi: 10.1378/chest.1679559.
54. Chen PN, Shih CK, Li YH, Cheng WC, Hsu HT, Cheng K. Gastric perforation after accidental esophageal intubation in a patient with deep neck infection. Acta Anaesthesiol Taiwan. 2014;52(3):143-5.
55. Consiglieri C, Gornals J, Huertas C, Madrazo Z, Loras C. Massive pneumoperitoneum during endoscopic ultrasound-guided drainage of a pancreatic cyst lesion, treated with an enteral self-expanding metal stent and paracentesis. Endoscopy. 2014; doi: 10.1055/s-0034-1377222.
56. Di Saverio S, Catena F. Rotation of the Liver in Pneumoperitoneum. N Engl J Med 2014; doi: 10.1056/NEJMicm1400032.
57. Montazeri М, Farhangi B, Montazeri M. Pneumatic Rapture of Rectosigmoid; a Case Report. Emergency. 2014;2(4):180-2.
58. Monterrubio-Villar J, Cordoba-Lopez A. Barotrauma during apnoea testing for brain death determination in a five-year-old boy.TheFreeLibrary.2014.http://www.thefreelibrary.com/Barotrauma+during+apnoea+testing+for+brain+death+determination+in+a...-a0188796909 Accessed 04 Jan 2015.
59. Shashidhara P, Shaziya HA, Seshasayi M. A case of tension pneumoperitoneum due to colonic barotrauma with compressed air. JEMDS. 2014; doi: 10.14260/jemds/2014/3530.
60. Ternlund SP, Brock-Utne JG. Failure to recognize tension pneumoperitoneum during resuscitation. The Free Library. 2014. http://www.thefreelibrary.com/Failure+to+recognize+tension+pneumoperitoneum+during+resuscitation.-a0188739762 Accessed 04 Jan 2015.
61. Thatte М, Taralekar SV, Raghuvanshi K. Colonic Barotrauma with Tension Pneumoperitoneum – Review of Literature and Report of A Successfully Treated Case. Int J Scient Res. 2014;3(4):339-41**.**
62. Webman R, Rosenzweig M, Bholat O, Bernstein M, Todd SR, Frangos SG. Tension pneumoperitoneum caused by blunt thoracic trauma. Trauma. 2014;16(1):56-9.
63. Williams D, Manoochehri P, Kim H. Images in emergency medicine: Tension pneumoperitoneum. Emerg Med J. 2014;31(11):943.
64. Chen YG, Chen JH, Huang TC.Tension pneumoperitoneum in a lung cancer patient with intestinal metastases. J Emerg Med. 2015;48(1):23-4.
65. Kaafarani H. Tension Pneumoperitoneum. N Engl J Med. 2015; doi: 10.1056/NEJMicm1500045.
66. Miller D. Tension Pneumoperitoneum Caused by Obstipation. West J Emerg Med. 2015;16(5):777-80.
67. Sy E, Chiu YI, Shan YS, Ong Rl. Pneumatic colon injury following high pressure blow gun dust cleaner spray to the perineum. Int J Surg Case Rep. 2015;6:218-21.
68. Thornton S, Hunter J, Scott M. Fatal Tension Pneumoperitoneum Due to Non-Accidental Trauma. West J Emerg Med. 2015; 16:788-9.
69. Vishnu RM, Laxman P, Sanjiv G. Development of pneumoperitoneum after CPR. Int J Surg Case Rep. 2015;6:40-2.
70. **Table S2** Cases of primary anaerobic bacterial peritonitis in healthy patients reported in the English medical literature.

| **Authors, year, ^[ref]^** | **Number of cases** | **Age and gender** | **Cultured bacterium** | **Outcome** |
| --- | --- | --- | --- | --- |
| Matthews P, 1979^[1]^ | 1 | 16-year-old boy | *Fusobacterium necrophorum* | Slow Full Recovery |
| Totten J, 1979^[2]^ | 1 | 16year-old girl | *Clostridium oedematiens* | Deceased |
| Elkassem S et al, 2008^[3]^ | 1 | 21 year-old woman | *Fusobacterium necrophorum* | Slow Full Recovery |

**Searched electronic libraries:** Pubmedcentral, Willey online library, Cochrane Library, Elsevier, The Free Library, Google Scholar, Internet Scientific Publications.

See below for the reference list of this research.

1. Matthews P. Primary anaerobic peritonitis. Br Med J. 1979;2(6195):903-4.
2. Totten J. Primary anaerobic peritonitis. Br Med J. 1979;2(6199):1225.
3. Elkassem S, Dixon E, Conly J, Doic C. Primary peritonitis in a young healthy woman: an unusual case. Can J Surg. 2008;51(2):40-1.
